# Supplementary material for: Integrative Analysis of Proteomics and Metabolism Reveals the Potential Roles of Arachidonic Acid Metabolism in Hypoxia Response in Mouse Spleen
Source: Molecules. 2022 Nov 21;27(22):8102. doi: 10.3390/molecules27228102 (PMC9696392; doi:10.3390/molecules27228102)
Supplement: Supplementary file 1 [file molecules-27-08102-s001.zip › molecules-1973056-supplementary.pdf]

Supplementary Figures

Integrative Analysis of Proteomics and Metabolism Reveals the Potential Roles of Arachidonic Acid Metabolism in Hypoxia Response in Mouse Spleen

Yujing Guo †, Sheng Yong †,*, Yuzhen Xu, Ying Hu, Jidong Li, Qifu Long, Xiaojun Wang, Cunlin Gu and Zengqiang Miao

Medical College of Qinghai University, Xining 810016, China
* Correspondence: yongsheng@qhu.edu.cn
† These authors contributed equally to this work.

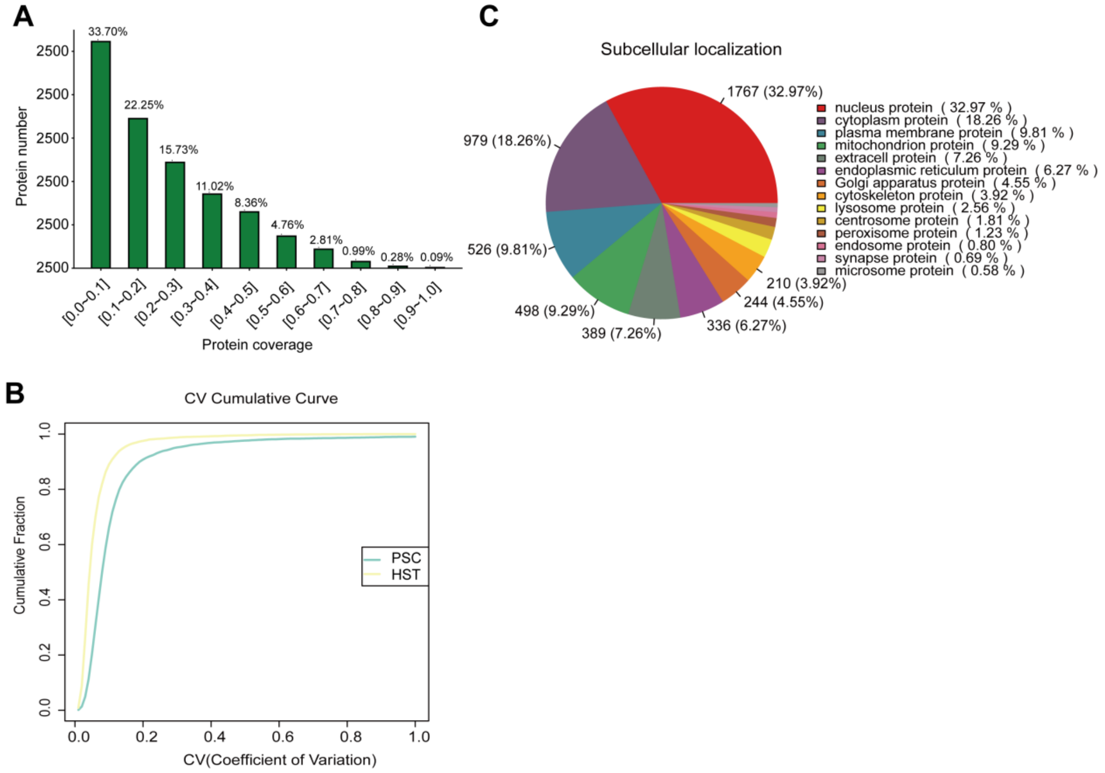

Figure S1. Protein identification and subcellular localization analysis **A**, Protein number distribution across different protein coverage mapped to peptides detected. **B**, Cumulative fraction analysis against the coefficient of variation in two groups. **C**, The subcellular localization analysis on the total 7727 proteins annotated based on proteomics analysis.

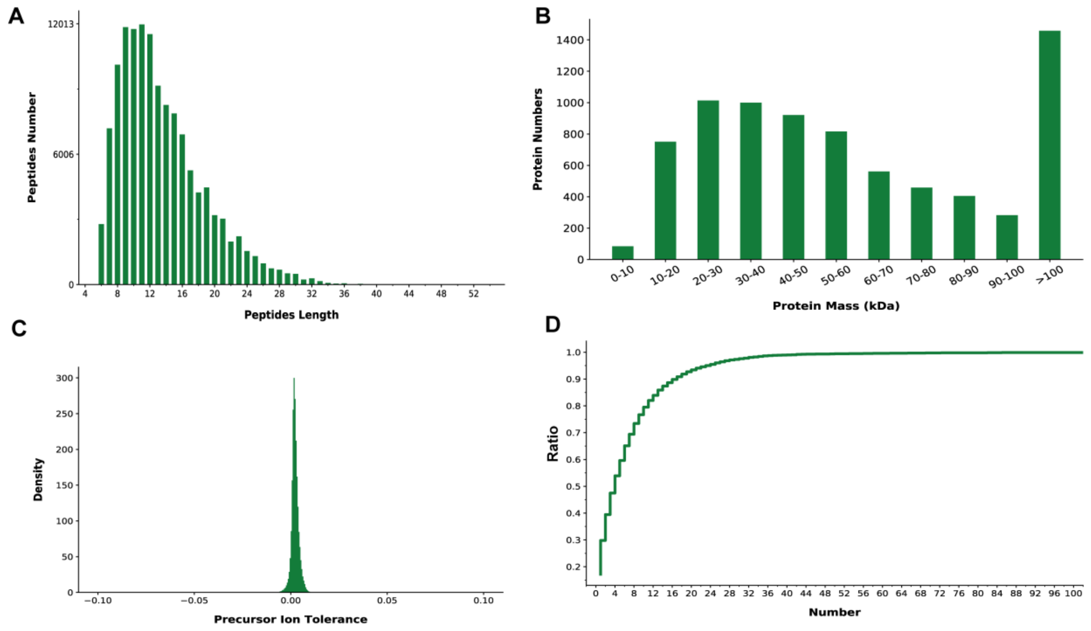

Figure S2. Quality control based on proteomic analysis. **A**, peptide analysis annotated across different peptides length. **B**, protein numbers distribution following different protein mass. **C**, histogram representing the density of mass spectrum signals along with different precursor ion tolerance. The numbers of total matched mass spectrum were 132,104. **D**, the ratio of unique peptides account for total peptides against different relative peptide numbers.

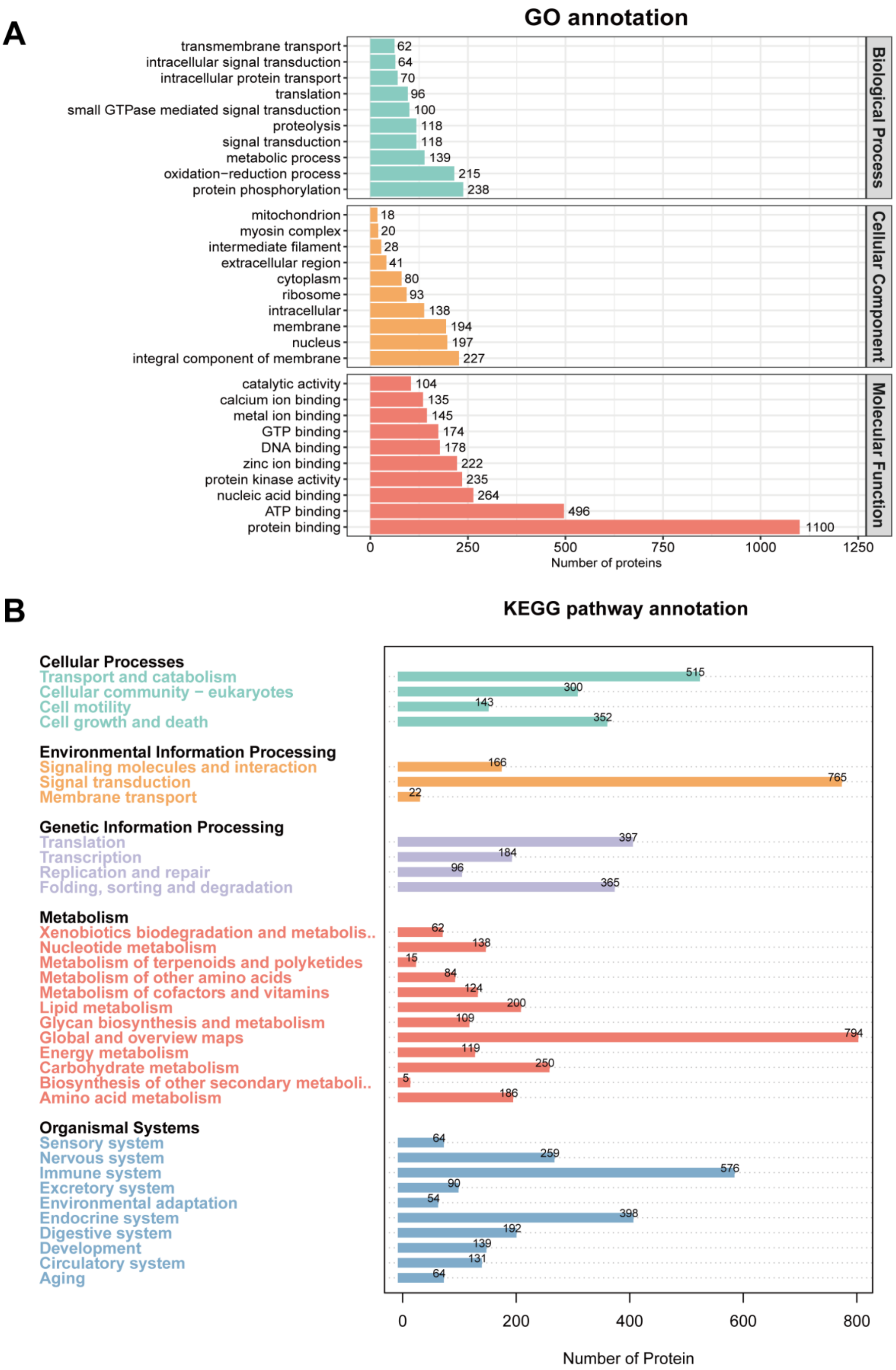

Figure S3. Bio-informatics analysis on the 7,727 protein annotated in mice spleen. **A**, Gene ontology (GO) analysis. **B**, Kyoto encyclopedia of genes and genomes (KEGG) analysis.

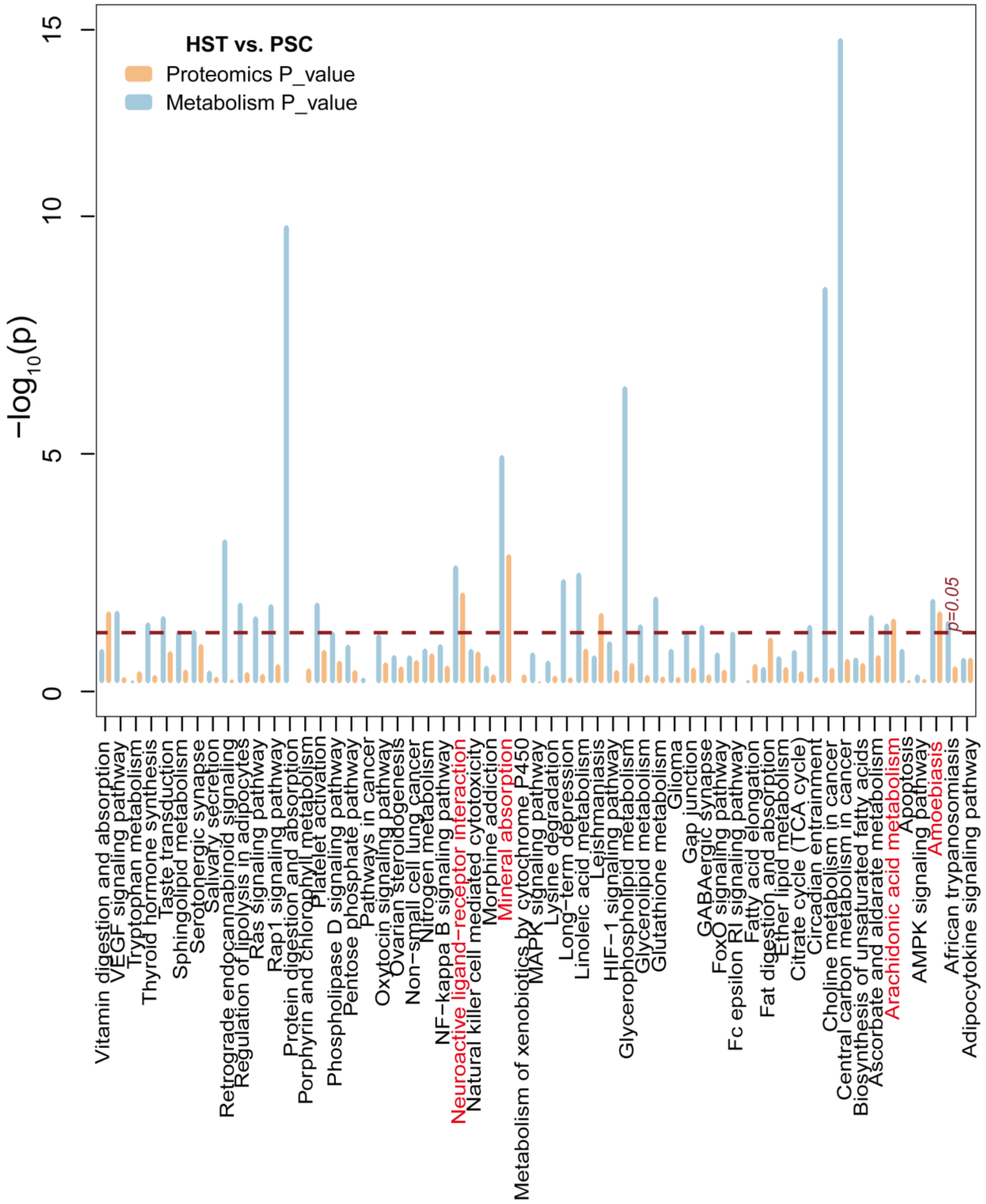

Figure S4. Overlapped KEGG terms on the list of differentially expressed proteins and differentially abundant metabolites. The dot line represents significant levels based on Fisher *t*-test at $P<0.05$. The red fonts represent the top 4 KEGG terms that referred to Figure 3C.

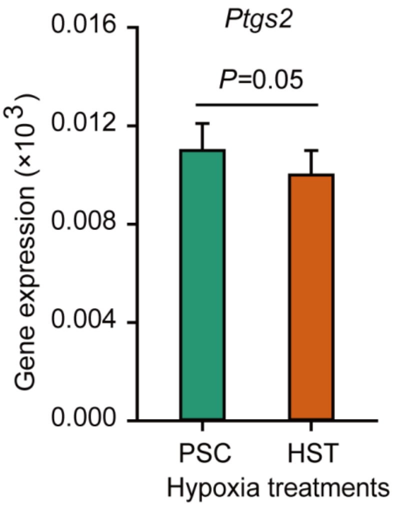

Figure S5. Relative expression levels of *Ptgs2* in spleen of mice exposed to either hypoxia or normaxia conditions. Each bar data represents the mean (±SE) of 3 different biological replicates.

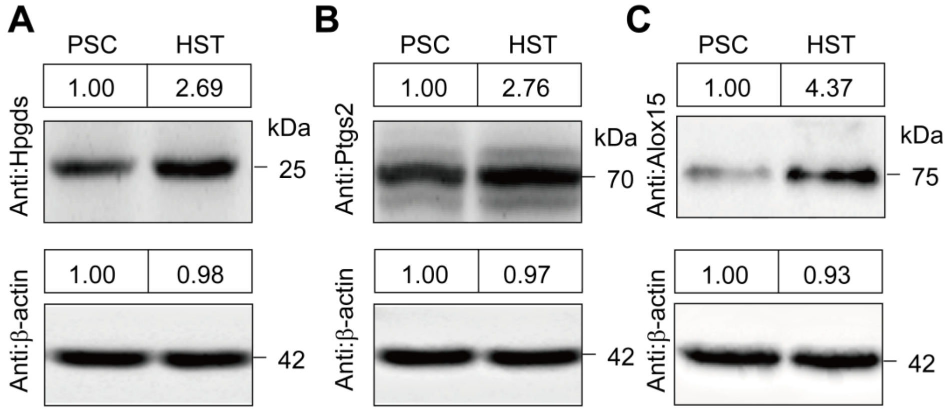

Figure S6. Western blot validation of two genes related to arachidonate acid metabolism in hypoxia-treated spleen of mice. **A**, Hpgds protein expression level. **B**, Ptgs2 protein expression level. **C**, Alox15 protein expression level. The numbers indicate the relative signal strength analyzed by Image J software. Antibodies of Hpgds and Ptgs2 were used to perform the western blot with at least three biological replicates.
